# Pericyte‐Assisted Vascular Lumen Organization in a Novel Dynamic Human Blood‐Brain Barrier‐on‐Chip Model

**DOI:** 10.1002/adhm.202401804

**Published:** 2025-05-06

**Authors:** Vita Guarino, Elisabetta Perrone, Elisa De Luca, Alberto Rainer, Maura Cesaria, Alessandra Zizzari, Monica Bianco, Giuseppe Gigli, Lorenzo Moroni, Valentina Arima

**Affiliations:** ^1^ Department of Experimental Medicine University of Salento Lecce 73100 Italy; ^2^ NANOTEC Institute of Nanotechnology Consiglio Nazionale delle Ricerche (CNR) Lecce 73100 Italy; ^3^ CBN Center for Biomolecular Nanotechnologies Istituto Italiano di Tecnologia (IIT) Arnesano 73010 Italy; ^4^ Department of Engineering Università Campus bio‐Medico di Roma Rome 00128 Italy; ^5^ Fondazione Policlinico Universitario Campus Bio‐Medico di Roma Rome 00128 Italy; ^6^ MERLN Institute for Technology‐Inspired Regenerative Medicine department of complex tissue regeneration Maastricht University Maastricht 6211 LK The Netherlands

**Keywords:** HCMEC/d3, bbb‐on‐chip, blood‐brain barrier, organ‐on‐chip, pulsating flow

## Abstract

Organ‐on‐Chip (OoC) technology provides a powerful platform for neurovascular research, enabling the precise replication of the blood‐brain barrier (BBB) microenvironment, including its 3D architecture and the influence of dynamic blood flow. This study introduces a novel microfluidic device designed to investigate the morphological and structural adaptations of human brain endothelial cells (ECs) within narrow, square‐shaped microchannels that closely mimic the microvessels of the brain's microcirculation. The endothelial microchannels are layered above a microchamber filled with Matrigel and abluminal vascular cells, enhancing cell‐cell interactions across the BBB interface. The system integrates co‐culture with pericytes and astrocytes while subjecting brain ECs to physiologically relevant pulsatile flow. The findings reveal that the morphology and cytoskeletal organization of brain ECs are distinctly influenced by pulsatile flow depending on the presence of pericytes and astrocytes. Specifically, in the absence of perivascular support, brain ECs exhibit a stretched morphology with prominent actin stress fibers, while co‐culture with pericytes and astrocytes promotes endothelial rearrangement, leading to lumen formation and enhanced barrier properties. This study highlights the essential role of perivascular cells in modulating endothelial responses under microvascular confinement and physiologically relevant flow. These insights advance in vitro models of the neurovascular unit and BBB mechanobiology.

## Graphical abstract

Organ‐on‐Chip (OoC) technology enables the precise modeling of the blood‐brain barrier (BBB). This study presents a microfluidic device that mimics brain microvessels, integrating co‐culture with pericytes and astrocytes under pulsatile flow. Results show that perivascular cells modulate endothelial morphology, promoting lumen formation and barrier integrity. These findings enhance neurovascular research and BBB mechanobiology.

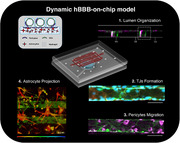

## 1 Introduction

Organ‐on‐chips (OoCs) are microfluidic‐based in vitro systems that are designed to replicate the functional unit of human organs on a miniature scale. The ultimate goal of OoCs is to provide a more realistic and human‐relevant environment for in vitro studies of cell and tissue activity and behavior, as well as to reduce the need for animal testing.^[^ ^1^, ^2^ ^]^

In the field of neurovascular research, OoCs give significant contributions by modeling the human blood‐brain barrier (hBBB) due to its peculiar structural features, which make the BBB more difficult to replicate than other vascular barriers.^[^ ^3^ ^]^ The architecture of the BBB is intricate and comprises various elements: i) the endothelial cells (ECs) that are closely connected through tight junctions (TJs) and form the wall of blood microvessels; ii) a basement membrane (BM), composed of extracellular matrix (ECM) proteins that surround the ECs; iii) pericytes that are contractile cells enveloping the ECs and enclosed within the BM; iv) astrocytes, a subtype of glial cells originating from the brain parenchyma that extend projections, known as end‐feet, making contact with and envelop the blood vessels. Together, these components create a highly selective barrier that allows essential nutrients, such as oxygen and glucose from the blood, to pass through. Additionally, they block the entry of most harmful substances, toxins, and pathogens. The BBB is in charge of protecting the delicate neural tissue and maintaining the homeostasis of the brain's microenvironment.^[^ ^4^, ^5^ ^]^

Significant attention has been directed toward utilizing OoC technology to explore the BBB functionality. The primary goals are emulating the 3D structure of the barrier and reproducing effects associated with blood flow dynamics, drug transportation across the barrier, and neurological disorders.^[^ ^6^, ^7^ ^]^ Current investigations on OoC‐based models of the BBB, known as BBB‐on‐chips, predominantly use steady flow conditions. These studies investigate the impact of various shear stress (SS) values falling within the physiological range (typically ranging from 1 to 95 dyn cm^−2^)^[^ ^8^, ^9^ ^]^ on barrier characteristics such as TJs expression, trans‐endothelial electrical resistance (TEER), and permeability coefficients.^[^ ^6^, ^7^, ^10^ ^]^

While assuming steady flows may serve to explore average responses, it is worth observing that in realistic conditions cerebral blood flow exhibits pulsatility, meaning changes over time, in all types of cortical microvessels, including capillaries and venules.^[^ ^11^, ^12^, ^13^, ^14^, ^15^, ^16^, ^17^, ^18^ ^]^ It is believed that brain capillaries have the ability to regulate and dampen the pulsatile nature of cerebral blood flow, enabling them to regulate the local neuronal activation by supplying additional nutrients and eliminating metabolic waste as needed.^[^ ^19^, ^20^ ^]^ Overall, the brain pulsatility is synchronized with the heartbeat, typically occurring at a frequency of the order of 1 Hz, and can lead to a dynamically changing temporal shear stress gradient (TSSG) experienced by brain ECs. Previous studies have indicated that a TSSG can have a more substantial impact on the ECs behavior than the absolute SS values.^[^ ^21^, ^22^, ^23^ ^]^ Furthermore, it is noteworthy that not only the presence of pulsatile flow but also its frequency level may influence the morphology and functions of ECs.^[^ ^24^, ^25^ ^]^ Pulsatility, especially when associated with elevated blood pressure or pressure peaks, or specific vascular irregularities, has the potential to negatively impact the blood vessels and tissues in the brain.^[^ ^11^, ^26^, ^27^ ^]^ These alterations may contribute to the development of cerebrovascular damage and diseases. Therefore, understanding the mechanisms governing cerebral blood flow pulsation, as well as its impact on blood vessels, can aid physicians in more accurately diagnosing and treating cerebrovascular conditions, ultimately leading to enhanced prognoses and improved patient quality of life.

This study introduces and investigates a novel hBBB‐on‐chip model, providing insights into how the BBB's cellular components react to a realistic pulsatile flow. Initially, the study assesses the impact of pulsatile flow on brain ECs cultured alone in the narrow, square microchannels, revealing that this kind of flow prompts cells to exhibit a stretched morphology with prominent actin stress fibers aligned in the flow direction. Then, ECs are co‐cultured with pericytes and astrocytes to develop the complete hBBB model. Our research highlights the roles of pericytes and astrocytes as regulators of vascular lumen organization and stability by influencing the orientation of actin filaments in ECs.

The findings presented herein demonstrate that our hBBB‐on‐chip faithfully replicates the intricate interactions among ECs, pericytes, and astrocytes under human‐relevant dynamic flow conditions, as well as  discloses the implications of the blood flow pulsatility on cell organization within microvessels. Finally, our hBBB model demonstrates reliable barrier permeability to reference molecules, suggesting its potential as a drug screening platform for central nervous system diseases.

## 2 Results and Discussion

### 2.1 A Novel Microfluidic Device Enhances BBB Modeling

**Figure 1:**
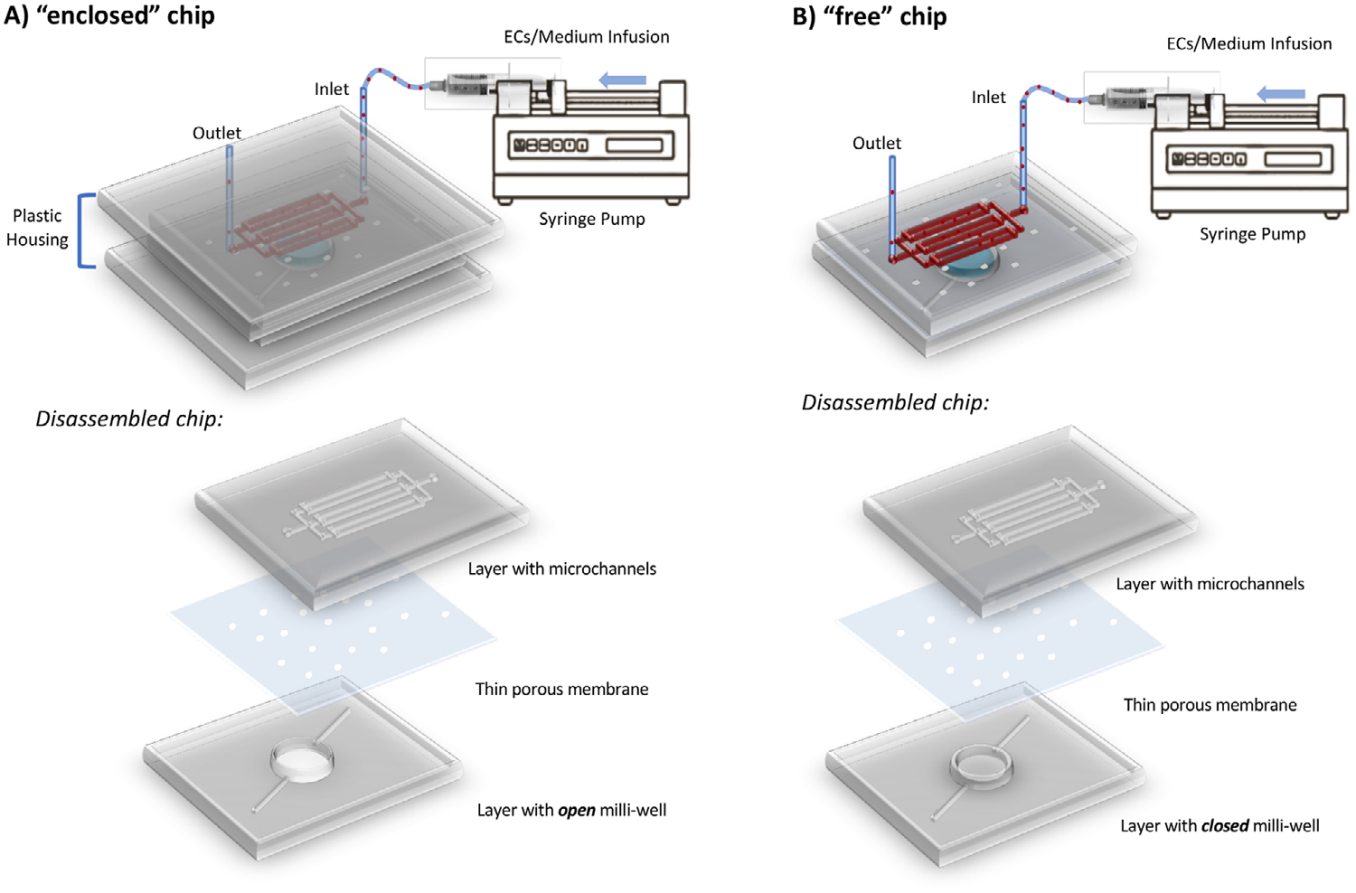
Schematic representation of two microfluidic chip designs: A) “enclosed” chip and B) “free” chip. Both configurations consist of a PDMS layer with microchannels, a thin porous PC membrane, and a second PDMS layer forming a milli‐well. In the “enclosed” chip, the milli‐well is open and sealed thanks to a plastic housing, while in the “free” chip, the milli‐well is closed, allowing for distinct experimental setups. The milli‐well represents the brain side of the hBBB model, enabling the static culture of pericytes and astrocytes, whereas the microchannels simulate the blood side, allowing brain ECs to be cultured under flow conditions.

Numerous BBB‐on‐chips, often fabricated from polydimethylsiloxane (PDMS), feature a dual‐layer design with a porous membrane sandwiched between an upper and lower layer. Typically, these devices contain only two channels, either running parallel or, less frequently, perpendicular to each other, with relatively small overlapping regions.^[^ ^7^ ^]^ In contrast, our PDMS‐based microfluidic chip presents a distinct innovation in its design. It incorporates a millimeter‐sized circular chamber (milli‐well), separated by a polycarbonate (PC) membrane with 8 µm diameter pores, from an array of square and straight microchannels (**Figure** **1**). Notably, our microchannels with a square cross‐section of  50 µm) are, to the best of our knowledge, the narrowest ever reported in a BBB‐on‐chip model, closely mimicking the dimensions of the microvessels in the brain's microcirculation.^[^ ^7^ ^]^

In this study, we developed two distinct dual‐layer chip designs, termed “enclosed” (Figure 1A) and “free” (Figure 1B), each offering specific advantages. The key difference between the two configurations lies in the milli‐well, specifically designed for the cultivation of pericytes and astrocytes, which can be either open or closed. The designation “enclosed” versus “free” reflects the necessity of embedding the chip within a plastic housing to seal the open milli‐well during dynamic flow experiments. In both chip designs, astrocytes embedded in a hydrogel were seeded and cultured under static conditions within the milli‐well, while pericytes were directly cultured on the membrane. Subsequently, the chips were inverted, positioning the microchannels on top, where brain ECs were seeded and cultured under continuous medium perfusion, delivered via silicone tubing connected to a syringe pump.

The primary advantage of the “enclosed” chip with an open milli‐well is its ease of cell seeding, viability testing, and conditioned medium collection (up to 50 µL). Moreover, this design minimizes air bubble formation, a common challenge in microfluidic devices, as the pressure within the microchannels gradually increases when filled with medium. A drawback of the “enclosed” chip is its overall thickness and the light‐scattering effect of the plastic housing, which restricts optical microscopy imaging during dynamic culture. Only after removing the holder—following cell fixation and staining—cellular organization can be assessed via light microscopy (end‐point optical studies). Conversely, the “free” chip, with a closed milli‐well, overcomes this imaging limitation by allowing direct perfusion without an external holder, making it compatible with live imaging microscopy during cell culture. However, its design presents challenges in cell and hydrogel seeding, as access to the milli‐well is only possible through side channels, increasing the likelihood of air bubble formation.

Overall, the two configurations are complementary and were selected based on the specific objectives of each experiment. Consequently, cell culture protocols were slightly adapted to accommodate the characteristics of each design.

As a further perspective, we aim to explore alternative materials or modifications to PDMS, as well as natural membrane substitutes, to better replicate the elastic properties of in vivo blood vessels.^[^ ^28^, ^29^, ^30^, ^31^ ^]^ These improvements are expected to enhance the physiological relevance of the model, enabling more accurate simulations of vascular function and responses under physiological conditions. The stiffness of PDMS microchannels can influence ECs mechanobiological responses, especially under dynamic flow, affecting how cells perceive and respond to SS and mechanical stimuli.^[^ ^32^, ^33^ ^]^ PDMS physical properties may lead to deviations from natural in vivo behaviors, impacting model accuracy for vascular studies. Additionally, addressing PDMS's inherent hydrophobicity is essential, as it can absorb small hydrophobic drugs, complicating its use in drug testing applications.^[^ ^34^ ^]^ Although PDMS offers advantages such as ease of fabrication, elasticity, optical transparency, and cost‐effectiveness, transitioning toward alternative materials for OoC fabrication is essential for developing more accurate and versatile in vitro models.^[^ ^31^ ^]^

While the microfluidic device herein presented remains a proof‐of‐concept, the current outcomes contribute valuable insights into less‐explored and debated aspects of the hBBB and its modeling. Overall, the device opens new avenues for studying the hBBB, encompassing investigations into brain ECs arrangement under pulsating flow, the involvement of pericytes and astrocytes in vascularization/endothelialization, and the measurement of barrier permeability to assess hBBB model functionality.

### 2.2 Brain ECs Exhibit Shear‐Induced Responses Under Pulsating Flow

The microchannels of our microfluidic device were designed with a narrow and square geometry, drawing inspiration from the studies of Tsai et al. and Inglebert et al., who modeled the lumen of microvessels.^[^ ^35^, ^36^ ^]^ Particularly noteworthy is the recent discovery by Inglebert et al. that human umbilical vascular ECs (HUVEC), which represent peripheral ECs, tend to detach from the corners of narrow square microchannels when exposed to low SS during prolonged steady flow. This effect results in the rounding of the engineered endothelial lumen.^[^ ^36^ ^]^

**Figure 2:**
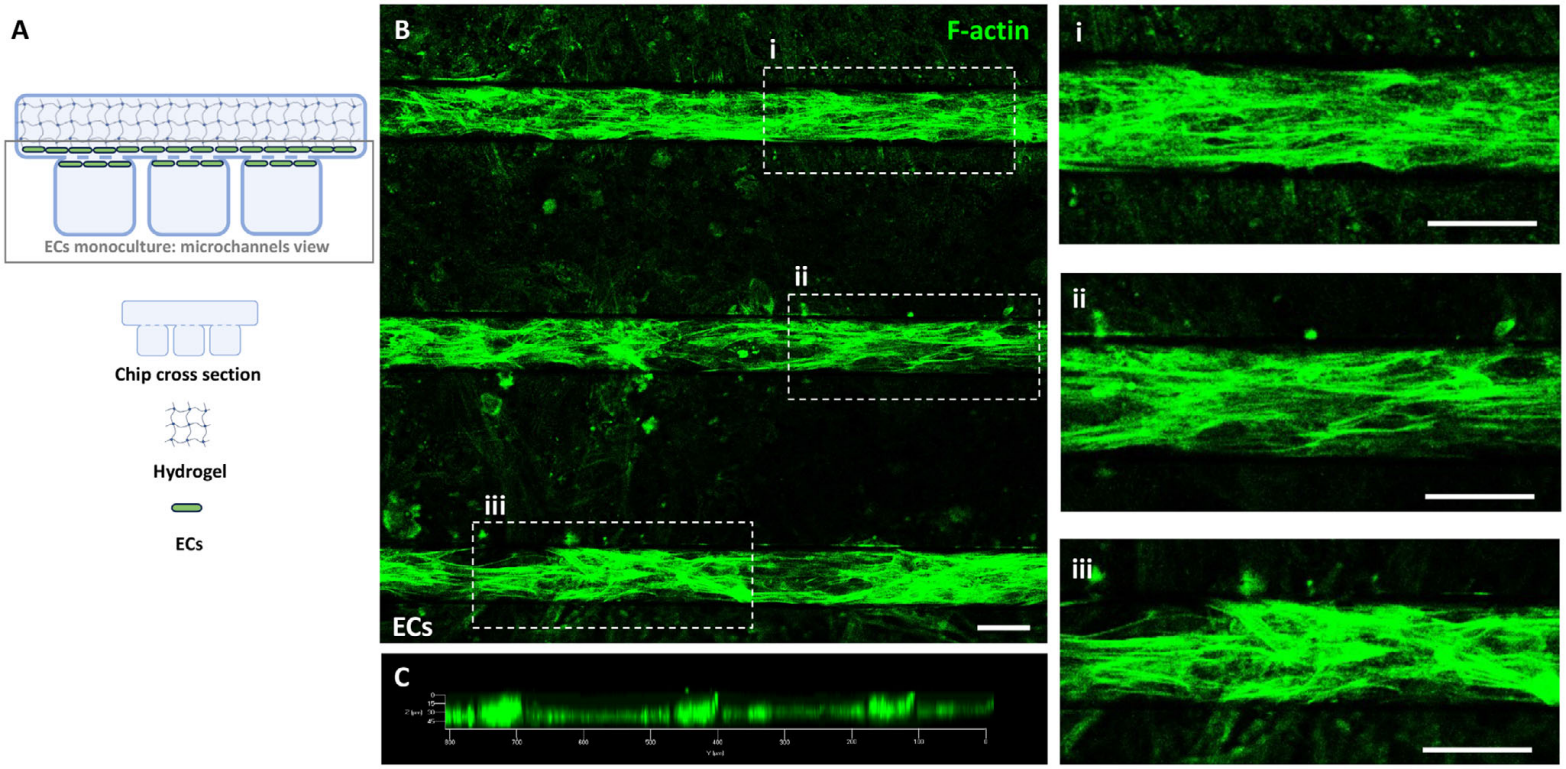
ECs monoculture in the microfluidic device. A) Schematic representation of ECs distribution within the chip, with the gray box indicating the region observed under confocal microscopy, referred to as “microchannels view.” B) Confocal image of F‐actin‐stained ECs within the microchannels, with insets i, ii, iii) showing magnified regions of cells featuring aligned, prominent actin stress fibers. C) 3D projections illustrate ECs adherence on both sides of the interposed membrane, with a tendency for alignment along the microchannel interface. (Scale bar: 50 µm).

In our investigation, we specifically examined the morphology and arrangement of human cerebral microvascular ECs (HCMEC/d3), which are an immortalized line representing human brain microvasculature ECs (hBMECs), within narrow square microchannels when exposed to low TSSG (a calculated time‐averaged value of 0.15 dyn cm^−2^) during prolonged pulsating flow with a physiologically‐relevant pulse frequency range (0.5–1 Hz).^[^ ^14^, ^17^ ^]^ After 72 h of culture, HCMEC/d3 cells in monoculture displayed distinct arrangement patterns (**Figure** **2**A), influenced by the initial seeding density (Figure S1, Supporting Information). Within the microchannels of the “enclosed” chip, HCMEC/d3 cells predominantly adopted an elongated morphology, with prominent actin stress fibers aligned along the direction of flow (Figure 2B). Cells showed a tendency to migrate toward the milli‐well and adhered to both sides of the interposed membrane, exhibiting a preferential alignment along the microchannel interface (Figure 2C).

The widely accepted perspective is that actin stress fibers contractility assists ECs in maintaining a flattened profile under dynamic flow conditions, preventing turbulence and effectively resisting fluid shear forces.^[^ ^37^ ^]^ Numerous studies have associated these morphological and cytoskeletal responses of peripheral ECs when exposed to absolute SS values within the physiological range.^[^ ^36^, ^38^, ^39^, ^40^, ^41^ ^]^ Our study demonstrates that flow‐induced responses are also observed in hBMECs when exposed to low TSSG, suggesting that pulsating flow can elicit responses similar to those induced in peripheral ECs by steady flow.

Previous investigations into the responses of hBMECs under dynamic flow conditions primarily concentrated on alterations in barrier properties (e.g., permeability coefficients, TEER), associated with barrier tightness and the expression of TJs.^[^ ^10^ ^]^ Few studies have specifically delved into the flow‐induced morphological and cytoskeletal responses of hBMECs, and consensus in this regard is yet to be reached (**Table** **1**).

**Table 1:** Orientation of hBMECs in response to dynamic flow conditions.

Ref.	Cell Type	Flow Type	Flow Timescale	SS/TSSG	Orientation
[42]	primary	steady	24 h	SS = 50 dyn cm^−2^	perpendicular to the flow
[43]	immortalized	steady	36 h	SS = 8‐12‐16 dyn cm^−2^	random
[44]	iPSC‐derived	steady	40 h	SS = 4–12 dyn cm^−2^	random
[45]	immortalized (HCMEC/d3)	steady	72 h	SS = 0.77‐3‐17 dyn cm^−2^	parallel to the flow
[46]	immortalized (HCMEC/d3)	steady	72 h	SS = 5–10 dyn cm^−2^	perpendicular to the flow
*This study*	immortalized (HCMEC/d3)	pulsating	72 h	TSSG = 0.15 dyn cm^−2^	parallel to the flow

Ye et al. were pioneers in observing that primary hBMECs, subjected to high SS (50 dyn cm^−2^ for 24 h) on the outside of 250 µm diameter glass rods, exhibited stress fibers oriented in various directions, with a notable prevalence of fibers aligned perpendicular to flow/rod direction.^[^ ^42^ ^]^ In contrast, HUVEC under the same conditions were predominantly aligned in the direction of flow. Ye's intriguing hypothesis proposed that ECs in the brain, unlike peripheral ECs, are inherently programmed to resist elongation in response to curvature and SS. This programming would aim to minimize the length of TJs per unit length of the capillary, consequently reducing paracellular transport into the brain.^[^ ^42^ ^]^ Conversely, both Reinitz et al. and DeStefano et al. demonstrated that both immortalized and induced pluripotent stem cell (iPSC)‐derived hBMECs (respectively), cultured under flow on glass bottom of rectangular channels, resisted elongation, and stress fibers remained randomly oriented under various conditions of physiological SS (8, 12, and16 dyn cm^−2^ for 36 h in Reinitz's work and 4 and 12 dyn cm^−2^ for 40 h in De Stefano's work).^[^ ^43^, ^44^ ^]^ It is noteworthy that in all these studies, the duration of flow exposure was less than 2 days, and comprehensive cytoskeletal remodeling and changes in cellular shape might necessitate considerably longer durations.^[^ ^47^ ^]^

Finally, Moya et al. and Choublier et al. explored the impact of physiological SS (0.77, 3, and 17 dyn cm^−2^ and 5 and 10 dyn cm^−2^, respectively) on the morphological and cytoskeletal reorganization of HCMEC/d3 over a minimum of 72 h of flow exposure.^[^ ^45^, ^46^ ^]^ However, their findings diverged. Moya et al. observed HCMEC/d3 alignment in the direction of flow with parallel actin filament orientation, coupled with significant morphological differences, especially more pronounced after 7 days of flow exposure compared to static conditions.^[^ ^45^ ^]^ On the other hand, Choublier et al. described cellular alignment perpendicular to the flow direction, aligning with the previously mentioned Ye's hypothesis.^[^ ^46^ ^]^ An important distinction in their experimental setups may account for these differing observations. In Choublier's study, HCMEC/d3 grew on glass bottom of rectangular channels (3.8 mm in width), while in Moya's study, HCMEC/d3 grew inside circular fibers (0.65 mm in diameter), exposing them to a more homogeneous SS.^[^ ^45^, ^46^ ^]^

In contrast to these previous studies, which applied steady flow and maintained constant SS values within the physiological range despite the pulsatile nature of cerebral blood flow,^[^ ^11^, ^12^ ^]^ our study presents a different approach. We continuously subjected HCMEC/d3 to pulsatile flow for at least 72 h and observed a distinct morphological response, with the cells exhibiting a stretched shape and prominent actin stress fibers aligned in the direction of flow. These findings challenge the assumption that hBMECs maintain a unique phenotype resistant to flow‐induced elongation and alignment. We demonstrated that under pulsatile flow, hBMECs not only elongated but also aligned parallel to the direction of flow, highlighting the critical role of TSSG in orchestrating this cellular response. By uncovering how hBMECs adapt to pulsatile flow, we provide new insights that refine the current understanding of brain EC  dynamics under physiological conditions. Future research will investigate how these responses are modulated by different flow environments (e.g., increased SS, varying frequencies), offering a deeper understanding of the mechanisms regulating endothelial function in cerebral blood flow and vascular health.

### 2.3 Pericytes Assist the Vascular Lumen Organization

**Figure 3:**
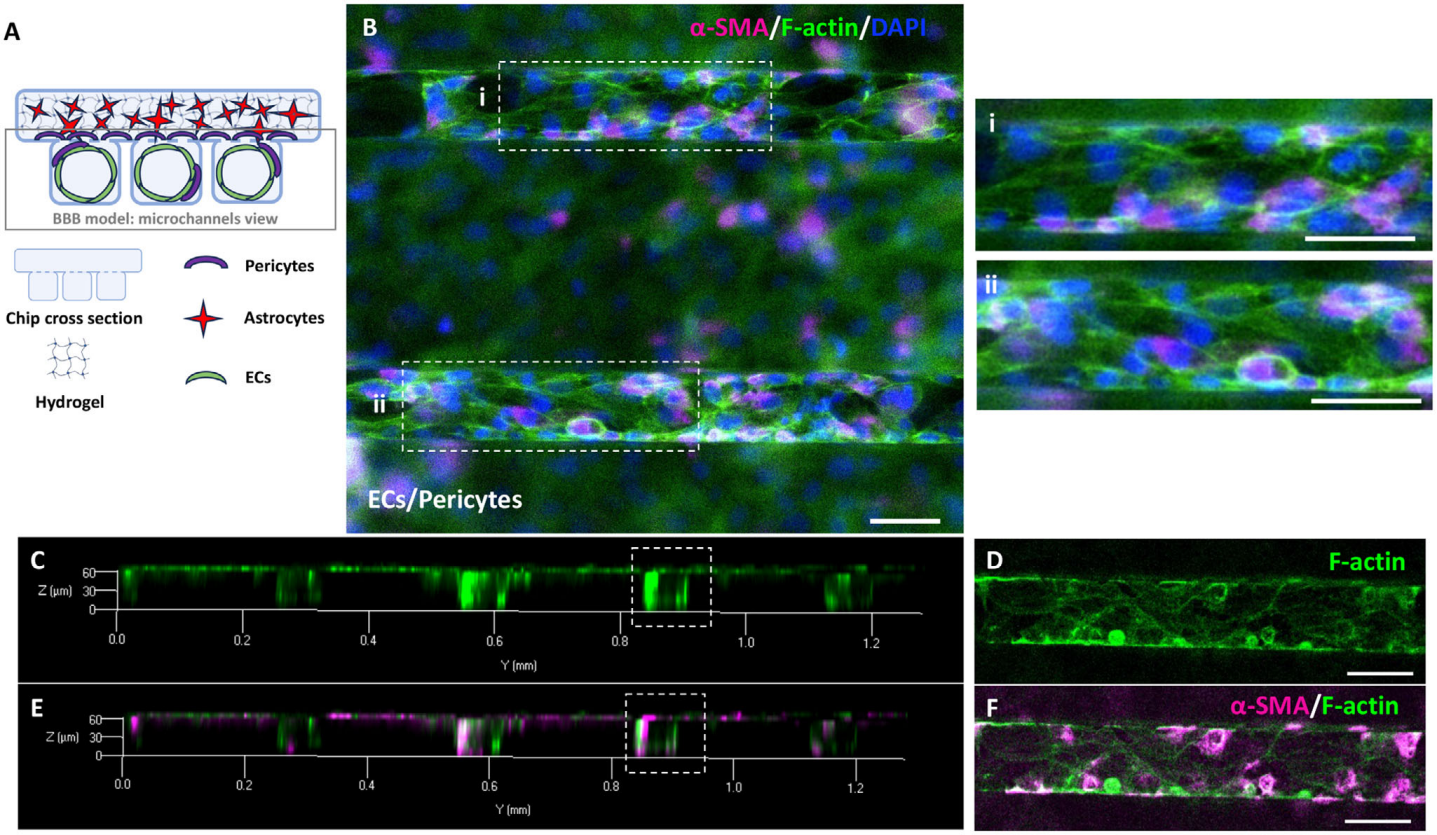
Vascular Lumen in the hBBB model. A) Schematic representation of ECs, pericytes, and astrocytes distribution within the hBBB model, with the gray box indicating the region observed under microscopy, referred to as “microchannels view.” B) Widefield image of microchannels in the hBBB model, with insets i, ii) showing magnified regions of walls lined by ECs partially covered by pericytes. Staining markers include Anti‐α‐SMA for Pericytes (magenta), DAPI for nuclei (blue), and FITC‐phalloidin for F‐actin (green). C) 3D rendering of confocal z‐stack highlights the uniform distribution of F‐actin (green) in cells along the entire surface of the microchannel walls, forming a well‐defined lumen, with a top view (D) on a single channel showing the organization of actin in the cortical area of cells. E) 3D rendering of confocal z‐stack highlights the distribution of α‐SMA‐stained pericytes (magenta), closely interacting with and partially surrounding the lumen within the microchannels, with a top view (F) on a single channel showing pericytes along the microchannels. (scale bar: 50 µm).

For the development of the hBBB model, HCMEC/d3 cells were introduced into the microchannels of the “enclosed” chip after Human Brain Vascular Pericytes (HBVPs) and Human Astrocytes (HAs) had been seeded in the milli‐well. The viability and features of HBVPs and HAs cultured in the milli‐well were separately investigated (Figures S2–S4, Supporting Information). Over the 72‐hour co‐culture period, during which pulsatile flow was applied, the organization of HCMEC/d3 within the hBBB model (**Figure** **3**A) exhibited a distinct arrangement compared to the monoculture condition in the microchannels, as previously described (Figure 2A).

In the hBBB model, HCMEC/d3 displayed a less elongated morphology with clearly defined cell‐to‐cell boundaries, where actin was organized into a cortical rim (Figure 3B). Remarkably, ECs formed a lumen with minimal presence of actin stress fibers (Figure 3C,D). Concurrently, HBVPs were observed migrating from the milli‐well to the microchannels, establishing direct contact with the endothelial lumen along the microchannels (Figure 3E,F). The push for migration of pericytes, induced by ECs, was indirectly validated through co‐seeding experiments involving HCMEC/d3 and HBVPs in the microchannels of the “free” chip, which enabled live imaging (see Supporting Information). Using cell‐specific tracers, we tracked the localization of HCMEC/d3, HBVPs, and HA over a 72‐hour culture period and observed that HCMEC/d3 and HBVPs remained confined within the microchannels, with no significant migratory behavior, and that HAs did not migrate from the milli‐well into the microchannels (Figure S5, Supporting Information).

Past research emphasized the crucial role of pericytes in orchestrating the formation of the BBB during embryogenesis.^[^ ^48^ ^]^ Pericytes also demonstrated the ability to regulate cerebral blood flow by detecting neuronal activity earlier than arterioles, actively relaxing to dilate capillaries.^[^ ^49^ ^]^ Thus, our findings led us to propose that the recruitment of pericytes by ECs played a pivotal role in organizing the endothelial lumen within the microchannels of our hBBB model. This involvement may have helped to mitigate fluctuations in SS over time induced by pulsatile flow. The mechanism of this regulation might have involved the release of signaling molecules by pericytes and changes in pericyte shape, which warranted further exploration in subsequent studies.

**Figure 4:**
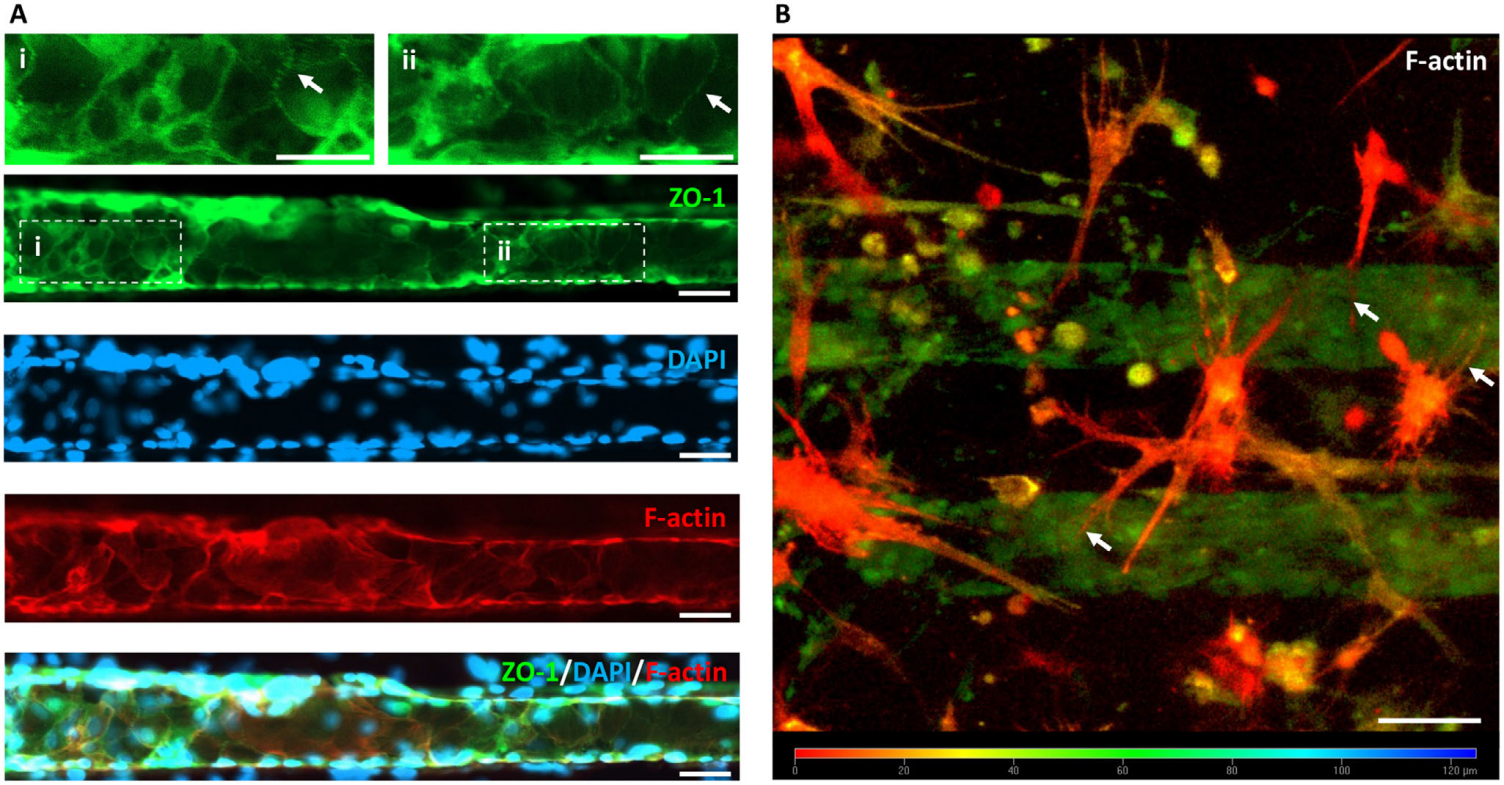
TJs and Astrocytic Projections in the hBBB Model. A) Confocal images of ECs within the microchannels stained for ZO‐1, DAPI, and F‐actin. Insets i, ii) highlight magnified regions where ZO‐1 displays a characteristic zipper‐like arrangement (white arrows), indicative of TJ organization. B) Depth map reconstructed from a confocal z‐stack of F‐actin‐stained cells, showing astrocytic projections (white arrows) extending from the milli‐well toward the microchannels, demonstrating their close spatial relationship with the endothelial lumen. (Scale bar: 50 µm).

Furthermore, while HCMEC/d3 monoculture within the microchannels primarily exhibited actin fibers extending throughout the cell body (Figure 2), their cytoskeletal organization underwent a significant shift in the presence of pericytes and astrocytes. Within the hBBB model, actin was predominantly rearranged into a cortical rim (Figure 3), a reorganization that correlated with endothelial lumen formation. Previous studies have highlighted the critical role of cortical actin in stabilizing cell‐cell adhesion complexes—TJs, adherens junctions, and desmosomes—thereby preserving the functional integrity of endothelial barriers.^[^ ^50^, ^51^, ^52^, ^53^ ^]^ Consistently, our results demonstrate that co‐culture with pericytes and astrocytes not only promoted endothelial lumen formation, but also led to TJ protein expression— particularly ZO‐1, which displayed a characteristic zipper‐like arrangement (**Figure** **4**A, Video S1, Supplementary Video 1).^[^ ^54^, ^55^, ^56^ ^]^

Lastly, the presence of HA extending their projections toward the endothelial lumen in our hBBB model was a key observation (Figure 4B). Previous studies have shown that astrocyte‐derived factors can induce BBB characteristics in ECs.^[^ ^57^, ^58^, ^59^ ^]^ Thus, we hypothesized that astrocytes contributed to endothelial lumen organization by releasing soluble factors—such as cytokines, growth factors, and ECM molecules—that likely stimulated endothelial remodeling and pericyte activation. Further investigations will be needed to characterize the specific factors released by astrocytes and their role in modulating endothelial and pericyte behavior in our model.

In conclusion, HCMEC/d3 monoculture within the microchannels exhibited a stretched morphology with prominent actin stress fibers aligned along the pulsatile flow direction. Conversely, in the hBBB model, HCMEC/d3 adopted a less elongated shape with fewer stress fibers, while actin filaments accumulated at the cortical borders, where ZO‐1 localized to support TJs. This cytoskeletal reorganization was closely associated with the formation of a well‐defined endothelial lumen. Additionally, HBVPs actively migrated from the milli‐well into the microchannels, establishing direct contact with the endothelial lumen, whereas HA remained confined to the milli‐well but extended long processes toward the microchannels. These findings suggest that pericytes provide structural support for vascular lumen formation, while astrocytes contribute essential signaling cues that facilitate this process.

To the best of our knowledge, vascular lumen formation has not been previously reported in any similar BBB‐on‐chip models.^[^ ^7^ ^]^ Even in designs with membrane pores sufficiently large to permit pericyte migration into the microchannels, these systems fail to provide the optimal size and geometry necessary to support microvessel formation.^[^ ^60^, ^61^, ^62^, ^63^ ^]^ In contrast, our microchannels were purposefully engineered to create the appropriate cellular confinement, facilitating lumen formation, consistent with previous studies on peripheral ECs.^[^ ^35^, ^36^ ^]^ However, when brain ECs were exposed to pulsatile flow in our microchannels as a monoculture, they did not form a tubular structure. Instead, co‐culturing with pericytes and astrocytes markedly influenced EC behavior, promoting the formation and organization of a vascular lumen in our hBBB model. Therefore, in our hBBB model, the SS experienced by brain ECs within the microchannels is spatially homogeneous, even though it fluctuates over time due to the pulsatile nature of the flow. This homogeneity arises from the flow being confined to a symmetrical region, ensuring a more uniform distribution of shear forces along the internal walls of the square microchannels, where the cells are evenly exposed on all surfaces. Other BBB‐on‐chip designs feature wide and shallow rectangular microchannels, where ECs predominantly cover the wall interfacing with the brain compartment.^[^ ^60^, ^61^, ^62^, ^63^ ^]^ In these configurations, flow distribution is uneven, with higher SS concentrated in the central region of the channel and lower stress near the edges, particularly along its width. This non‐uniform flow exposure can significantly impact barrier integrity, potentially leading to heterogeneous cellular responses and compromising the physiological relevance of the model.

Thus, our hBBB‐on‐chip model successfully replicates a physiologically relevant microvascular structure, also enhancing the spatial homogeneity of SS across brain ECs, thereby providing a more accurate simulation of in vivo conditions. We simulated the cerebral flow pulsatility and provided new insights into flow‐induced endothelial responses. While previous studies have primarily focused on the roles of pericytes and astrocytes in modulating the expression and formation of TJ proteins in ECs of the BBB, our research highlights their critical involvement in the morphological and cytoskeletal reorganization of brain ECs. Looking ahead, future research will explore the effects of various flow regimes to further understand their impact on endothelial behavior and BBB function.

### 2.4 Actin Filaments Alignment in ECs Drives the Lumen Organization

**Figure 5:**
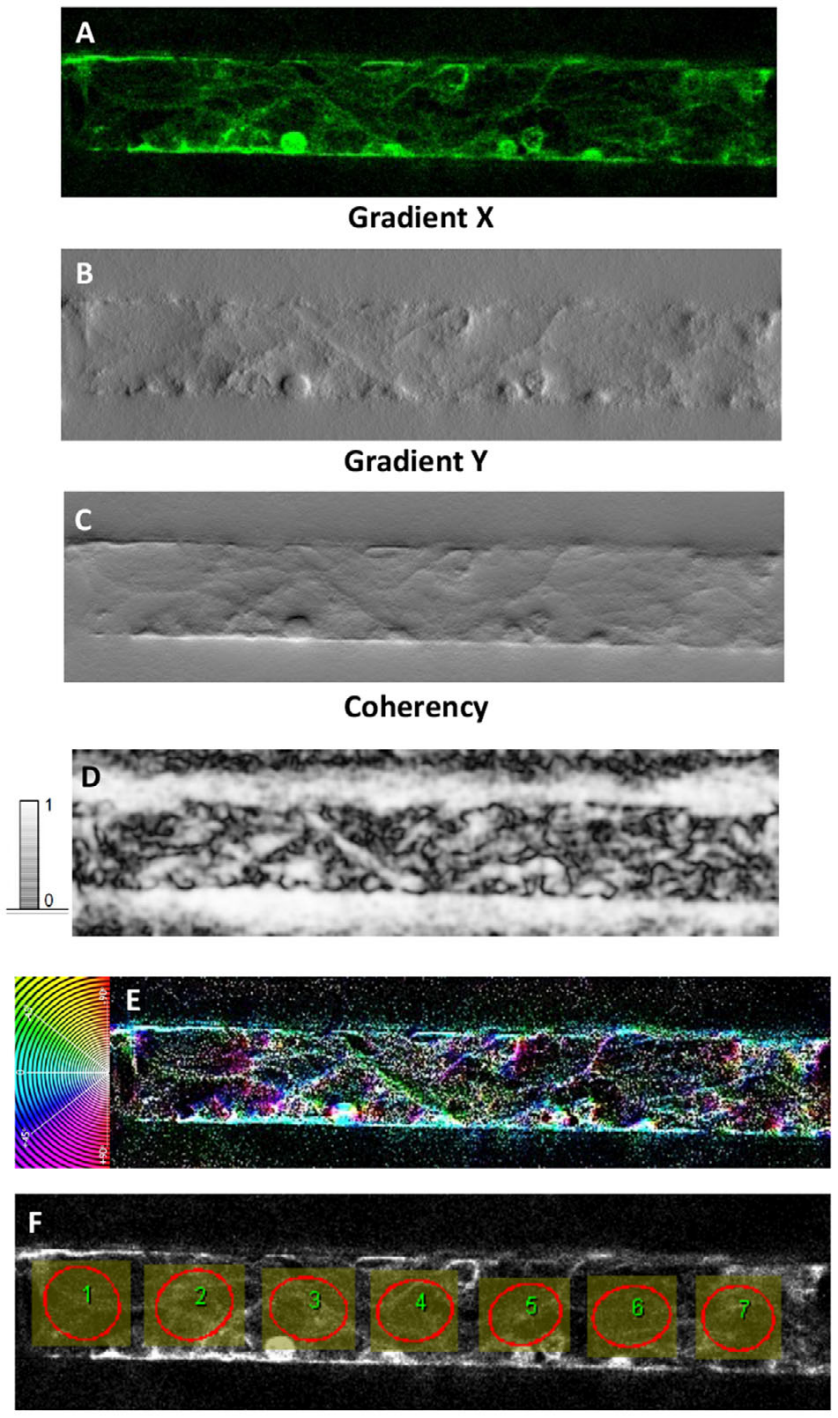
OrientationJ‐based analysis of HCMEC/d3 in the hBBB model. A) Source image. B) Gradient X image. C) Gradient Y image. D) Coherency map. E) Hue–saturation–brightness (HSB) color‐coded map together with the color wheel. F) Local distribution of the orientations investigated by the ellipse measure method.

**Figure 6:**
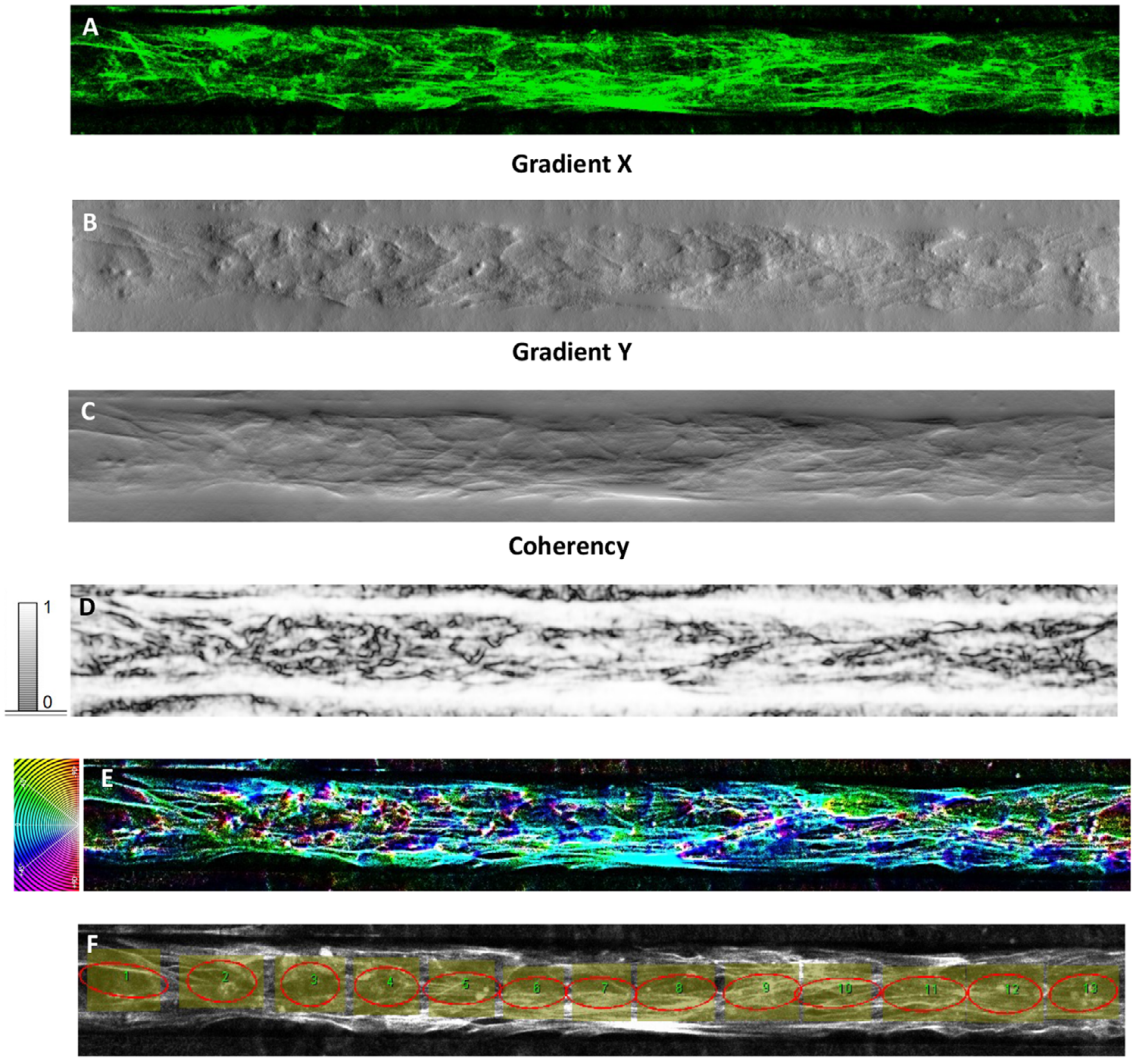
OrientationJ‐based analysis of HCMEC/d3 monoculture with microchannels. A) Source image. B) Gradient X image. C) Gradient Y image. D) Coherency map. E) Hue–saturation–brightness (HSB) color‐coded map together with the color wheel. F) Local distribution of the orientations investigated by the ellipse measure method.

OrientationJ was exploited to quantify the directionality of actin filaments within the cytoskeleton in fluorescent images of HCMEC/d3 exposed to pulsating flow, as well as to deeply investigate alignment/arrangement differences between HCMEC/d3 co‐cultured with HBVPs and HAs in the hBBB model and HCMEC/d3 within microchannels as a monoculture (**Figure** **5**A and **6**A, respectively). As an additional comparison, the OrientationJ‐based analysis assessed the orientation distribution characteristics of HCMEC/d3 cultured on a standard coverslip under static conditions (Figure S6, Supporting Information).

The structure gradient maps show the occurrence of different local textures, well highlighted by the gradient X (Figure 5B and 6B) and Y images (Figure 5C and 6C). The coherency images (Figure 5D and 6D) point out the occurrence of globular local structures (low coherency values) and locally elongated fibers that are associated with maximum coherency (C = 1, white color) and correspond to local specific orientations in the color‐coded HUE maps (Figure 5E and 6E). In order to map the occurrence of local alignment/ordering related to inherent cell reorganization, the ellipse method was applied to the inner region of the microfluidic wall in such a way as to rule out from the analysis the effect of the direction θ = 0° associated with the horizontal walls of the channel (Figure 5F and 6F). In this respect, contiguous equally sized and shaped ROIs were set along the full length of the channel. The ROIs, conforming to the areas circumscribed by ellipses, were kept as large as possible to allow the examination of the cell orientations without relevant effect from cell bundles and channel walls as well as small enough to let a statistical analysis of the local orientations along the channel and on accounting of the micrometer‐scale of the cell size. Moreover, these ROIs were carefully selected to exclude regions where pericytes were in contact with ECs in the case of the hBBB model (Figure S7, Supporting Information). By focusing on these specific areas, we ensured that the actin alignment analysis was primarily quantifying the filaments of ECs, minimizing the potential influence of pericyte actin filaments.

Consistently with the information provided from the structure gradient (Figure 5B,C), ellipses in Figure 5F are poorly elongated that is they exhibited more circular‐like than elongated elliptic shape, meaning no preferential cell alignment locally. This approach enabled us to assess that, in the hBBB model, poor general cell alignment developed along the horizontal direction in the case of HCMEC/d3 co‐cultured with pericytes and astrocytes.

On turning to the single HCMEC/d3 monoculture within microchannels (Figure 6), the textural features pointed out by the gradient maps (Figure 6B,C) clearly an increased degree of cell alignment compared to HCMEC/d3 in the hBBB model (Figure 5). This statement is confirmed by the distribution of coherency (Figure 6D): white elongated regions are evident within the channel region, meaning ordering along the horizontal direction. Consistently, the orientation‐coded colored map (Figure 6E) shows a global alignment trend of the cells. As the elongation of the ellipse is the quantitative measure of the image's local alignment and ellipses collectively are elongated around the direction θ = 0°over the entire length of the channel (Figure 6F), image analysis clearly demonstrates that cell alignment of the HCMEC/d3 monoculture was higher than cell ordering of the HCMEC/d3 in the hBBB model.

Therefore, the results from the structure tensor‐based orientation analysis provided quantitative support for our earlier hypothesis: astrocytes and pericytes influence the cytoskeletal organization of brain ECs in the hBBB model, reducing the impact of pulsating flow observed in the HCMEC/d3 monoculture. This results in a different arrangement of brain ECs along the microchannel walls, promoting the formation and stability of the vascular lumen.

### 2.5 Assessment of BBB Model Indicates Functional Barrier Integrity

**Figure 7:**
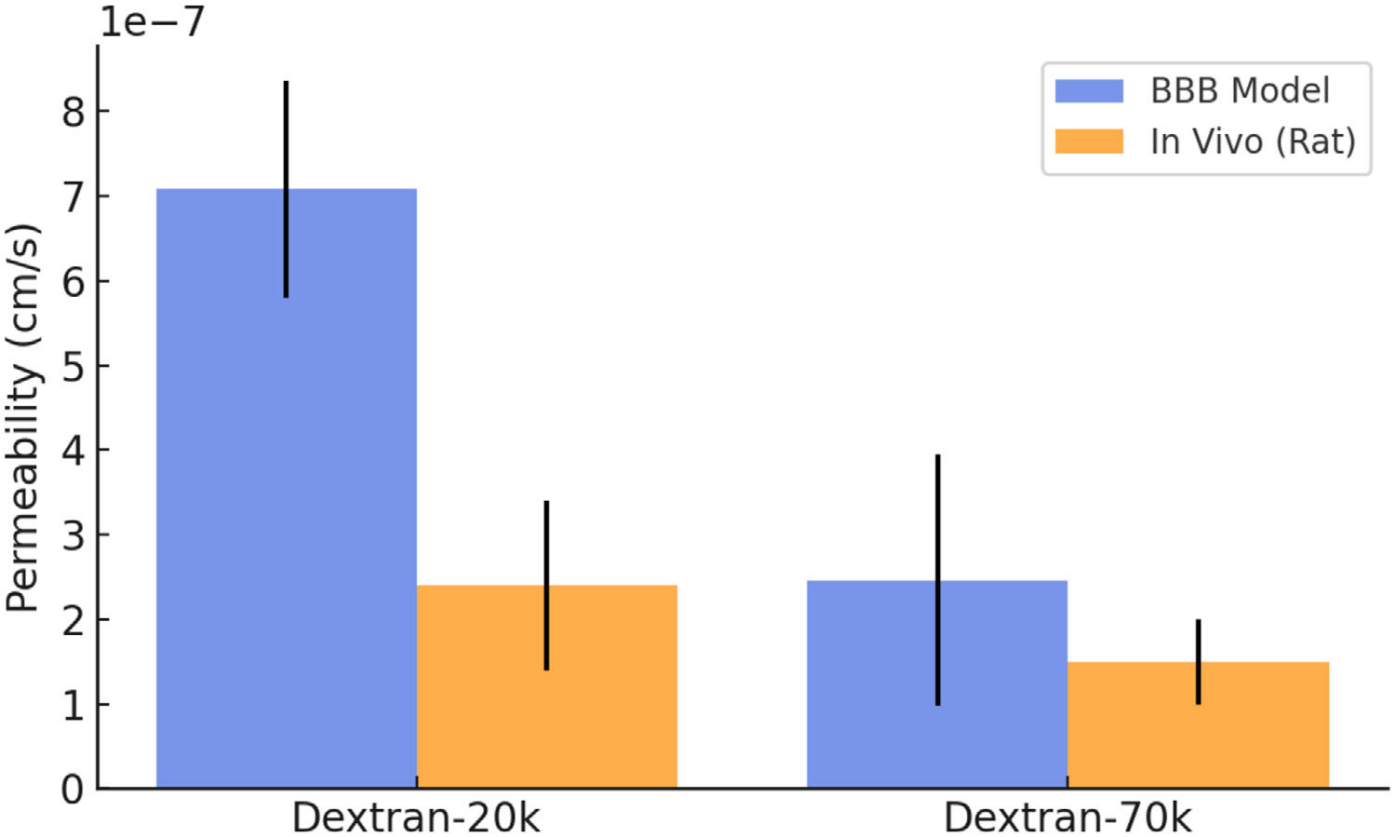
Permeability Comparison:BBB Model versus In Vivo. The apparent permeability coefficients (Papp (cm ^−1^s)) of our hBBB model (blue bars) closely align with those measured in rat brains (orange bars) for Dextran‐70K and Dextran‐20K, demonstrating comparable permeability within the same order of magnitude.

To evaluate the barrier function of our hBBB model, we performed permeability assays and TEER measurements. The permeability coefficients for 20 and 70 kDa FITC‐Dextran were assessed by sampling the medium collected from the milli‐well after overnight perfusion in the microchannel layer. This allowed us to maximize the accumulation of the tracer in the static milli‐well compartment. Fluorescence quantification of the collected medium enabled the calculation of the permeability values, which were (7.08 ± 1.28)×10⁻⁷ cm ^−1^s for 20 kDa FITC‐Dextran (*n* = 3) and (2.46 ± 1.48)×10⁻⁷ cm ^−1^s for 70 kDa FITC‐Dextran (*n* = 3), respectively. These values were consistent with in vivo rat brain permeability measurements ((2.4 ± 1)×10⁻⁷ cm ^−1^s for Dextran‐20k and (1.5 ± 0.5)×10⁻⁷ cm ^−1^s for Dextran‐70k) (**Figure** **7**).^[^ ^64^ ^]^

Furthermore, live‐imaging‐based permeability assessment is ongoing to achieve a more accurate assessment of the barrier's permeability, in particular for low molecular weight reference molecules (Figure S8, Supporting Information). These experiments will help refine our model, validating its potential as a future drug screening platform.

The TEER values of our hBBB model were calculated by measuring resistance by a custom reading system (Figure S9, Supporting Information), yielding a normalized value of 9.1 ± 2.8 Ω cm^2^ at the end of the 72‐hour co‐culture period (*n* = 3). This is significantly lower than the in vivo TEER values typically observed in rat brain vessels, which average ≈1500 Ω cm^2^.^[^ ^65^, ^66^ ^]^ However, it is essential to recognize that resistance measurements are subject to variability and are influenced by structural disparities between in vivo and in vitro models. Additionally, factors related to OoCs design, such as cell type, presence or absence of a matrix, applied SS, electrode type, and other considerations, contribute to this variability.^[^ ^67^, ^68^, ^69^, ^70^ ^]^ Despite this, our resistance measurements proved reliable for comparing different experimental conditions (Figure S10, Supporting Information). Therefore, TEER can be a valuable tool for cross‐validating permeability coefficients across various setups.

## 3 Conclusion

The study presented herein introduces a novel hBBB‐on‐chip model that offers novel insights into how the cellular components of the BBB respond to the stimulus of a pulsatile flow, which is a phenomenon inherent in cerebral blood flow.

Two distinct dual‐layer chip designs, “enclosed” and “free,” were developed to offer specific advantages for cell culture experiments. The main difference between the two is the milli‐well, which can either be opened or closed, depending on whether the chip is embedded in a plastic housing. Both designs feature astrocytes cultured in a hydrogel within the milli‐well, while pericytes are cultured on the membrane. The chips are inverted for brain ECs seeding within microchannels. The “enclosed” chip facilitates easy cell seeding and medium collection but is limited by optical imaging due to its thickness and light scattering. The “free” chip, while better suited for live imaging, poses challenges in seeding and increased air bubble formation.

Our research begins by examining the flow‐induced effects on brain ECs cultured alone within narrow and square microchannels. The findings demonstrate that subjecting brain ECs to pulsating flow, which generates a continuous and prolonged exposure to a TSSG, leads to stretched cell morphology and prominent actin stress fibers aligned in the direction of flow, forming a distinct arrangement along the interface within the microchannels. This cellular phenotype reflects the cells' adaptation to maintain a flattened profile under dynamic flow conditions, minimizing turbulence and effectively resisting fluid shear forces.^[^ ^37^ ^]^ These findings challenge the previous assumption that brain ECs possess a unique phenotype resistant to flow‐induced SS.^[^ ^42^, ^43^, ^44^ ^]^

The study then broadens its scope by developing a more comprehensive hBBB model, co‐culturing brain ECs with pericytes and astrocytes to replicate the cellular complexity of the hBBB. The results demonstrate significant differences in the cytoskeletal organization, morphology, and spatial arrangement of brain ECs in this co‐culture model compared to the monoculture within the microchannels, highlighting the influence of the surrounding cell types on endothelial behavior. In the BBB model, ECs establish intimate cell‐to‐cell contacts characterized by primarily cortical actin borders, TJs formation, and minimal actin stress fibers, leading to the formation of a well‐defined vascular lumen surrounded by pericytes. Concurrently, astrocytes extend their processes toward the microchannels, likely enhancing the structural and functional integrity of the barrier through essential signaling mechanisms. This spatial arrangement reflects a coordinated interaction among the cells, resembling the native architecture of the hBBB, where pericytes and astrocytes are essential for maintaining vascular stability and supporting endothelial function.

To the best of our knowledge, this is the first among BBB‐on‐chip models based on a templating approach (predetermined architecture) that reports the formation of a vascular lumen with size and shape close to the in vivo hBBB, and that also investigates the pulsating flow.^[^ ^7^, ^60^, ^61^, ^62^, ^63^ ^]^ These results not only advance our understanding of the hBBB's physiology, but also offer a robust platform for investigating diverse aspects of the intricate interaction among brain ECs, pericytes, and astrocytes. Finally, permeability studies conducted on our hBBB model yielded permeability coefficients comparable to physiological values, indicating the model's fidelity in replicating barrier properties. A comprehensive characterization of permeability will be a key focus in the continued validation of this model.

This work not only paves the way for investigating the hBBB's role in neurological disorders and brain‐targeted drug delivery but also provides a foundation for designing novel therapeutic strategies to enhance or protect barrier function in disease. Future technological advancements, such as the integration of real‐time biosensors within the chip, could enable live monitoring of barrier integrity markers and dynamic cell responses. Additionally, material innovations could improve biomimicry by better replicating the elasticity and mechanical properties of in vivo blood vessels, further refining the model's physiological accuracy.

## 4 Experimental Section

### Design and Fabrication of Microfluidic Device

The microfluidic device was designed to get a double‐layer layout consisting of a layer with a milli‐well (8 mm in diameter, internal volume of 100 µL) overlapped to another layer consisting of a pattern of 32 multiple straight mutually parallel microchannels with a square cross‐section (50 × 50 µm), length l = 8 mm, spacing s = 50–200 µm. The microfluidic device was fabricated with PDMS (Sylgard 184; Dowsil) by using soft lithography. To obtain the PDMS slab of the microchannels layer, PDMS pre‐polymer (10:1 elastomer base to curing agent, wt/wt) was degassed and poured onto silicon wafers patterned via optical lithography with SU‐8 2075 (Micro resist technology) for 15 min at 140 °C. Instead, to obtain the PDMS slab of the milli‐well layer, PDMS pre‐polymer was poured onto a Digital Light Processing (Asiga Max) printed master for 1 h at 65 °C. After curing, the microchannel layer was modified with two inlets and two outlets using a 1.5 mm biopsy punch, while the milli‐well was opened by removing the upper wall. A PC membrane (8 µm pore, track‐etched; Whatman) treated for 20 min at 80 °C with 5% 3‐aminopropyl‐trienthoxysilane aqueous solution (Sigma‐Aldrich/Merk) was sandwiched and bonded between the PDMS layers using a plasma cleaner (Diener electronic).^[^ ^71^ ^]^ Before the cells were seeded, the PDMS chip underwent a 30‐minute UV sterilization process. During perfusion experiments, the “enclosed” PDMS chip was positioned within a custom transparent plastic holder, manufactured by micromilling (MF70, Proxxon). Alternatively, the “free” version of the PDMS chip retained the upper PDMS wall of the milli‐well, facilitating perfusion without the holder and enabling live imaging.

### Cell Cultures

HCMEC/d3 (Cederlane) were cultured in EndoGRO‐MV medium (Millipore Merk) or alliteratively in Ham's F12 (Sigma‐Aldrich/Merk) supplemented with 5% Fetal bovine serum (Sigma‐Aldrich/Merk), 5% L‐Glutamine (Sigma‐Aldrich/Merk), 0.25% Penicillin (10000 IU mL^−1^) and Streptomycin (10000 µg mL^−1^) (Sigma‐Aldrich/Merk), 1 µg mL^−1^ Hydrocortisone 21‐hemisuccinate sodium salt (Sigma‐Aldrich/Merk), 50 µg mL^−1^ L‐ascorbic acid (Sigma‐Aldrich/Merk), 0.75 U mL^−1^ Heparin (Sigma‐Aldrich/Merk), 1 ng mL^−1^ human Basic Fibroblast Growth Factor (Sigma‐Aldrich/Merk). For all experiments, HCMEC/d3 was used between passages 29 and 33. HBVPs (Sciencell) and HAs (Sciencell) were cultured in astrocyte and pericyte medium, respectively (Sciencell). Both primary cells between passages three and five were used for all experiments.

### Cell Growth into the Microfluidic Device

At the beginning of culture time, the PDMS chip was placed with the milli‐well layer facing upward, and the PC membrane was coated with 100 µg mL^−1^ fibronectin by incubating it for 1 h at 37 °C. After that, HBVPs were seeded into the milli‐well with a density of 1.7 × 10^5^ cells cm^−2^ and incubated for 3 days in static conditions. Then, 1.7 × 10^6^ cells mL^−1^ of HAs were embedded in a Matrigel solution to fill the milli‐well. The Matrigel solution was prepared as a 1:1 dilution of the Matrigel Growth Factor Reduced stock (Corning) with astrocytes medium and the volume of the HA suspension was 10% of the final volume. After the Matrigel gelation in the milli‐well by incubating at 37 °C for 45 min, the astrocyte medium was added to prevent the gel from drying out. After overnight static incubation, the chip was inverted (with the microchannels layer faced up) and placed in its dedicated holder to proceed with HCMEC/d3 seeding and growing in dynamic conditions. The device was connected to a syringe pump (Model NE‐4000 Multi‐Phaser by KF Technology) and a solution of 50 µg mL^−1^ fibronectin bovine plasma (Sigma‐Aldrich/Merk) was injected into the microchannels. The device was placed for 1 h at 37 °C to allow the fibronectin self‐assembly on the microchannel walls. HCMEC/d3 were then seeded into the microchannels with a density of 1.5–1.8×10^6^ cells mL^−1^. The cell suspension was prepared by suspending the cell pellets in the medium added with 8% of Dextran from *Leuconostoc spp*. Mr 450 000–650 000 (Sigma‐Aldrich/Merk), previously filtered with a 0.22 µm filter. The dextran addition in the cell medium increases the viscosity of the cell suspension and hence the cell residence time in the microchannels, thus promoting a uniform cellular coating.^[^ ^35^ ^]^ The device underwent a 2‐hour incubation period in static conditions to facilitate cellular adhesion to the microchannel walls. Subsequently, fresh medium was infused into the device over a 3‐day period. During this time, HCMEC/d3 cells were cultured within the microchannels using the lowest syringe pump flow rate (1.5 µL h^−1^) to induce pulsating flow. The variations in flow rate over time within the microchannels were quantified by using a commercially available flow sensor (Figure S11, Supporting Information), measuring the occurrence of pulsating flow within a frequency range of 0.5–1 Hz. The introduction of a time‐varying flow rate condition led to the formation of a TSSG, yielding a calculated time‐averaged value of 0.15 dyn cm^−2^. To calculate the time‐averaged SS (𝜏𝑠 = dyn cm^−2^) applied during the dynamic culture of ECs, the following formula was used:

(1) $$\tau s=\frac{6\mu Q}{h^{2}\cdot w}$$

where *μ* was the viscosity of the cell culture medium;^[^ ^45^, ^72^ ^]^ *Q* is the inlet flow rate set on the syringe pump; *h* and *w* refer to height and width, respectively, of the microchannels.

### Cell Staining and Imaging

For imaging analysis of the hBBB model at the end of the culturing period, fixation was carried out with 3.7% Formaldehyde (Sigma‐Aldrich/Merk) for 15 min. Then, permeabilization was achieved with 0.1% Triton‐X100 (Sigma‐Aldrich/Merk) for 15 min, followed by blocking with 1% BSA (Invitrogen) for 1 h. All reagents were flowed both layers of the PDMS chip using a peristaltic pump (REGLO Digital from Ismatec). After blocking, cells were exposed to the primary mouse antibody anti‐αSMA (1:100 in 1% BSA; R&D Systems) at 4 °C overnight. The following day, a quick PBS wash was performed before introducing the secondary donkey antibody anti‐mouse Alexa Fluor 647 (1:500 in 1% BSA; Invitrogen) for 1 h at room temperature. For filamentous actin (F‐actin) and nuclei staining, TRITC/FITC‐phalloidin (0.05 µg mL^−1^, Sigma‐Aldrich/Merk) and DAPI (0.1 µg mL^−1^, Sigma‐Aldrich/Merk) were injected under continuous flow for 10 min each. Finally, PBS was used to wash the cells before imaging.

In the live imaging experiments (see Supporting Information), three distinct cell tracers were used: Vybrant CFDA SE Cell Tracer (1:2000, Invitrogen) for monitoring HCMEC/d3, CellTracker Red CMTPX dye (1:2000, Invitrogen), and CellTracker Deep Red dye (1:1000, Invitrogen) for tracking HAs and HBVPs, respectively.

Moreover, immunostaining was conducted to verify the expression of GFAP in HAs embedded in Matrigel and the expression of ZO‐1 in HCMEC/d3 within microchannels. Antibodies used for these experiments were the primary rabbit antibody anti‐GFAP (1:100 in 1% BSA; Invitrogen), the secondary goat antibody anti‐rabbit Alexa Fluor 555 (1:1000 in 1% BSA; Invitrogen), and the primary mouse antibody anti‐ZO‐1 monoclonal (ZO1‐1A12) conjugated with Alexa Fluor 488 (1:100 in 1% BSA; Invitrogen). Finally, a live/dead kit (Invitrogen) was employed for the viability assays (refer to Supporting Information).

Images were acquired by a widefield microscope (EVOS M7000, Thermos Fisher) and confocal microscopes (Zeiss LSM 700 and LSM 980) at 10× magnification. In particular, z‐stacks were acquired with the confocal microscopes exciting the samples with the excitation lasers at 488, 555, or 639 nm. Z‐stacks were collected using optimal intervals.

### Permeability Assay

The permeability assays were conducted to assess the endothelial barrier integrity of the hBBB‐on‐chip with labeled tracers. After 3 days of dynamic culture, the endothelial medium was replaced by medium added with 1 mg mL^−1^ of 20 or 70 kDa FITC‐dextran (Sigma‐Aldrich/Merk), which was perfused overnight through the microchannels layer to maximize the accumulation of the tracer accumulation in the static milli‐well compartment. After overnight perfusion, medium from the milli‐well was collected and FITC‐dextran was detected and quantified by using a Plate Reader (CLARIOstar Plus–BMG LABTECH). The measured fluorescence values were used to calculate the apparent permeability coefficient (P_app_ = cm ^−1^s) as follows:

(2) $$Papp=\frac{C_{brain}}{C_{blood}}\cdot \frac{flowrate}{Area}$$

where, *C_brain_* refers to the concentration of FITC‐dextran in the milli‐well, calculated after the accumulation period with the measured fluorescence intensity values using a standard calibration curve; the quantity *C_blood_* is the concentration of the FITC‐dextran in the medium flowing through the microchannels layer at the constant *flow rate* of 1.5 µL h^−1^ during the accumulation period; *Area* was calculated as the total value of the endothelial surface area covering the microchannels sides overlapped to the milli‐well (A = 0.128 cm^2^).

### TEER measurement

The endothelial barrier's TEER was assessed using a customized system featuring electrode wires positioned at the termination of the inlet and outlet channels (see Supporting Information). Ag, Ag/AgCl electrode wires (0.5 mm in diameter; Word Precision Instruments) were connected to an EVOM3 volt‐ohmmeter (Word Precision Instruments). This volt‐ohmmeter generates a consistent 10 µA of alternating current at 12.5 Hz while simultaneously gauging resistance. To derive the TEER values (Ω cm^2^), the recorded resistances were multiplied for endothelial surface area (A = 0.128 cm^2^) and then normalized by subtracting the baseline resistance of the chip without cells (Matrigel‐only conditions).

### Cell Orientation Analysis

Quantitative information on the orientation and isotropy/anisotropy properties of the actin‐stained cells imaged by confocal microscopy was obtained by digital image processing. The automated plugin OrientationJ (EPFL) of the license‐free software ImageJ/FIJI was used to calculate the structure tensor matrix around a given point (x_0_,y_0_) of the image.^[^ ^73^ ^]^ The 2D structure tensor associated with a given function of two variables f(x,y), is defined as follows:

(3) $$J{\left(P_{0}\right)}_{w}=\int \;\;\;\int w\left(P-P_{0}\right)\left(\begin{array}{cc} f_{x}^{2}\left(P\right) & f_{x}\left(P\right)f_{y}\left(P\right)\\ f_{y}\left(P\right)f_{x}\left(P\right) & f_{y}^{2}\left(P\right) \end{array}\right)\mathrm{dxd}y$$

where w(x, y) ≥ 0 is a weighting function associated with a ROI around P_0_ = (x_0_,y_0_) and fk is the partial derivative of f(x,y) with respect to the variable k (k = x, y). Local orientation and anisotropy of an image can be addressed by the angle θ (i.e., the maximum eigenvalue of the structure tensor), the energy E (i.e., the trace of the structure tensor), and the coherency index C (ranging from zero to unit, where C = 1 means occurrence of a dominant orientation and C = 0 means isotropic image) defined as follows:^[^ ^73^, ^74^ ^]^

(4) $$\theta =\frac{1}{2}\;\mathrm{arctg}\;\left(2\frac{{\langle f_{x}|f_{y}\rangle }_{w}}{{\langle f_{y}|f_{y}\rangle }_{w}-{\langle f_{x}|f_{x}\rangle }_{w}}\right)$$

(5) $$E={\langle f_{x}|f_{x}\;\rangle }_{w}+{\langle f_{y}|f_{y}\rangle }_{w}={\left(gradX\right)}^{2}+{\left(gradY\right)}^{2}$$

(6) $$C=\frac{\sqrt{{\left({\langle f_{y}|f_{y}\rangle }_{w}-{\langle f_{x}|f_{x}\rangle }_{w}\right)}^{2}+4{\langle f_{x}|f_{y}\rangle }_{w}}}{{\langle f_{x}|f_{x}\rangle }_{w}+{\langle f_{y}|f_{y}\rangle }_{w}}=\frac{\lambda_{\max}-\lambda_{\min}}{\lambda_{\max}+\lambda_{\min}}$$

where the weighted inner product <f|g>w is given by the integral:

(7) $${\langle f|g\rangle }_{w}=\int \;\;\;\int w\left(x,y\right)f\left(x,y\right)g\left(x,y\right)\mathrm{dxdy}$$

and λ_max_ and λ_min_ (λ_max_ ≥ λ_min_ ≥ 0) are the maximum and minimum eigenvalues of the structure tensor.

The source image is processed based on the associated histogram of the gray‐level intensity: two principal spatial directions x and y are set to calculate the structure tensor, and a Gaussian‐shaped window with size set by the user (the default value is 0.5 pixels) is translated over the entire image and the gradient operator is calculated according to different options (cubic spline, finite difference gradient, finite difference Hessian, Fourier gradient, Gaussian gradient, and Riesz filter). Riesz filter operator was the choice because it is a translation‐, rotation‐, and scale‐invariant transformation able to reduce the image noise and avoid the amplification of high frequencies. OrientationJ provides a visual orientation representation, with the orientation encoded in a HUE color visualization (hue‐saturation‐brightness map where HUE is orientation, saturation is coherency, and brightness is the input image). Moreover, for quantitative local investigation of orientational features, the ellipse measure mode was applied. Once an ROI is selected, the measure mode looks for local dominant orientation and draws an ellipse defined by three parameters that are direction, size, and elongation (ratio of major to minor axes). Elongation is related to coherency that takes into account the largest eigenvalue (major axis) and the smallest eigenvalue (minor axis). Hence, vanishing coherency (minimum) is associated with an ellipse being a circle, which is an absence of elongated structure (local orientation) in the ROI of the image. Maximum coherency C = 1 is associated with an ellipse becoming a line segment, which is a perfectly elongated structure (dominant local orientation) in the position of the image under consideration.

It is worth observing that the analysis involving the structure tensor is very easy and robust, it overcomes the complications and inaccuracy related to image binarization, which is ruled out, and benefits from a proper choice of the functional (the Riesz filter operator in the case) to control/reduce the impact of the acquisition‐related noise and contrast of the image as well as different weights of the frequencies in image processing. Moreover, the structure tensor method avoids several pitfalls of the Fast Fourier Transfer (FFT) method, which commonly works in the presence of strong periodic behavior and well‐evident directions.

### Statistical Analysis

All experiments were performed at least three times or repeated in three batches of independent experiments. Data were presented as the mean ± standard deviations (SD).

## Conflict of Interest

The authors declare no conflict of interest.

## Supporting information



Supporting Information

Supplemental Video 1

Supporting Information

## Data Availability

The data that support the findings of this study are available from the corresponding author upon reasonable request.
