# Supplementary material for: Pericyte‐Assisted Vascular Lumen Organization in a Novel Dynamic Human Blood‐Brain Barrier‐on‐Chip Model
Source: Adv Healthc Mater. 2025 May 6;14(15):2401804. doi: 10.1002/adhm.202401804 (PMC12147984; doi:10.1002/adhm.202401804)
Supplement: Supplementary file 1 — Supporting Information [file ADHM-14-0-s001.docx]

Supporting Information

**Pericyte-Assisted Vascular Lumen Organization in a Novel Dynamic Human Blood-Brain Barrier-on-Chip Model**

*V. Guarino^*^, E. Perrone, E. De Luca, A. Rainer, M. Cesaria, A. Zizzari, M. Bianco, G. Gigli, L. Moroni, V. Arima*

^*^Email: [vita.guarino@unisalento.it](mailto:vita.guarino@unisalento.it)

1. **Brain Endothelial Cells (ECs) within the microchannels**

In the “enclosed” chip, we investigated the morphology and arrangement of human cerebral microvascular ECs (HCMEC/d3) as a monoculture, when exposed to low TSSG (a calculated time-averaged value of 0.15 dyn/cm^2^) during prolonged pulsating flow with a physiologically-relevant pulse frequency range (0.5 – 1 Hz).

At the end of the culturing period, cell arrangement exhibited distinct patterns corresponding to the seeding density. In conditions with approximately 1 x 10^6^ cells/mL, cells partially covered the microchannel walls but were uniformly distributed (Figure S1-A,a). Conditions with 1.5–1.8 x 10^6^ cells/mL resulted in a complete coverage, but exclusively along the interface wall between microchannels and milli-well layers (Figure S1-B,b). Finally, conditions with over 2 x 10^6^ cells/mL meant cell death.

It is crucial to emphasize that our chip incorporated a porous interfacing membrane that prompted the migration of several ECs from the microchannels to the milli-well when they are as a monoculture. Therefore, increasing the cell seeding density affected the cell propension to migrate towards the milli-well and to adhere above the membrane. This behavior can be attributed to ECs receiving more nutrients from the medium within the milli-well than the medium flowing within the thin microchannels. Indeed, without flow, viable cells were exclusively present in the milli-well, and not in the microchannels.

**Figure S1. Confocal images of F-actin-stained HCMEC/d3 within the microchannels.** Under condition of seeding density of 1 x 10^6^ cells/mL (A), elongated ECs partially covered the microchannel surfaces in the image, while the 3D rendering of a confocal z-stack (a) illustrates homogeneous cell adhesion across all microchannel walls and on the membrane from the milli-well side. Under condition of seeding density of 1.5–1.8 x 10^6^ cells/mL (B), the microchannel surfaces in the image were fully covered by ECs showing prominent actin stress fibers, but the 3D rendering of a confocal z-stack (b) indicates that the majority of cells were concentrated at the interfacing walls and on the membrane from the milli-well side. (Scale bar: 100 µm).

1. **Astrocytes and Pericytes into the milli-well**

The behavior of astrocytes and pericytes was individually investigated in the microfluidic device under static conditions, excluding the influence of brain ECs. Initially, live/dead assays were conducted to verify the viability of these cells within the device before the seeding and growth of brain ECs (Figure S2 and S3).

Notably, pericytes exhibited morphological heterogeneity (Figure S2-A and S2-B), a characteristic commonly observed in these cells cultured *in vitro* (1). A pericyte pattern with retraction of projections was evident at a low seeding density (Figure S2-C and S2-D), closely resembling the pattern observed *in vivo* for migrating pericytes within microvessels (2). Additionally, the pericytes were evenly distributed across the porous membrane in the milli-well (Figure S2-B), with no observed preference for migration toward the microchannels, in contrast to the behavior seen in the presence of brain ECs within the microchannels.

Regarding astrocytes, their arrangement within the Matrigel filling the milli-well was uniform and multilayered, displaying a characteristic interconnected network architecture and star-shaped morphology (Figure S3 and S4-A). Furthermore, astrocytes exhibited the expression of glial fibrillary acidic protein (GFAP), a classical marker indicative of the main processes of mature astrocytes (Figure S4-B).

**Figure S2.** Live/Dead Assay on a Monoculture of Pericytes. Cell viability was evaluated after 3 days of culture within the milli-well of the device, preceding astrocytes seeding. (A) The image depicts both alive (green) and dead (red) pericytes within the milli-well of the device, with a seeding density of 1.7 x 10^5^ cells/cm^2^. (B) The image emphasizes the distribution of pericytes on the porous membrane in comparison to the microchannels below. (C) The image illustrates alive (green) and dead (red) pericytes within the milli-well of the device, with a seeding density of 1 x 10^5^ cells/cm^2^. (scale bar = 100 μm) (D) A magnified view shows a morphological subtype of pericytes exhibiting a retraction of projections. (Scale bar = 100 µm (A – C), scale bar = 50 μm (D)).

**Figure S3.** Live/Dead Assay on a Co-culture of astrocytes and pericytes. Cell viability was examined after 24 hours of co-culture. (A) The image exhibits both alive (green) and dead (red) cells within the milli-well of the device, with astrocytes being more discernible than pericytes due to their upper location. (B) The image highlights the cellular distribution within the milli-well in contrast to the microchannels below. (Scale bar = 100 μm).

**Figure S4.** Astrocytes Embedded in Matrigel. Astrocytes were cultured as a monoculture for 7 days within the milli-well of the device filled with Matrigel. (A) Live astrocytes captured in a phase-contrast image (scale bar = 100 μm). (B) Fixed astrocytes imaged under fluorescent microscopy, labelled for GFAP (red) (Scale bar = 100 μm).

1. **Live imaging of the BBB model development**

To enable live imaging experiments, a "free" version of the PDMS chip was designed, meaning an unenclosed chip placed in a holder (Figure 1B, Main Text). This “free” chip configuration retained the upper wall of the milli-well, allowing perfusion while facilitating real-time imaging. In contrast, the "enclosed" version of the chip (Figure 1A, Main Text) hindered live imaging due to the thickness and light-scattering properties of the plastic holder.

For the development of the BBB model using the "free" chip, we refined the cell seeding protocol as follows: astrocytes, encapsulated in Matrigel, were seeded first. After Matrigel polymerization, ECs and pericytes were seeded in the microchannels at a 1:2 ratio. To track the three cell types, we employed distinct cell tracers (refer to Materials and Methods, Main Text) and captured fluorescence images immediately after seeding (T0) and after 72 hours of dynamic culture in the incubator (T1).

As shown in Figure S5, astrocytes (red) remained confined within the Matrigel-filled milli-well over time, while ECs (green) and pericytes (magenta) were restricted to the microchannels. Only a small number of cells migrated to the milli-well, adhering to the membrane after 72 hours. This allowed us to confirm that pericytes preferentially localize near brain ECs, demonstrating that their migratory behavior is influenced by the presence of brain ECs. In fact, when pericytes and brain ECs were seeded separately, as in the "enclosed" version of the chip, pericytes actively migrated toward the microchannels, where they accumulated along and around the vascular lumen (see Results and Discussion, Main Text).

**Figure S5. Fluorescence images of the BBB model at T0 (immediately after seeding) and T1 (72 hours of dynamic culture).** ECs (green) (A, E) and pericytes (magenta) (B, F) are predominantly localized within the microchannels, while astrocytes (red) remain confined within the Matrigel in the milli-well (C, G Compared to the overlay of the initial seeding stage (D), minimal migration of ECs and pericytes into the milli-well and membrane adhesion is observed in the overlay of 72 hours of culture (H). (Scale bar: 100 µm).

1. **Orientation Analysis of HCMEC/d3 under static conditions.**

We employed the OrientationJ plug-in within ImageJ/FIJI to assess the orientation distribution characteristics of HCMEC/d3 cultured on a standard coverslip under static conditions until confluency (Figure S6). Directional gradient maps along the horizontal x-direction and vertical y-direction are presented in Panels B and C, respectively. Panel D displays a color-coded image illustrating the cell arrangement for a visual representation of orientations distributed according to the color wheel. Panel E features an intensity polar plot depicting the local orientation distributions of cells. Notably, the polar plot indicates the absence of a dominant orientation in this case, as it encompasses all orientations with some spikes.

**Figure S6. Orientation J-based analysis of HCMEC/d3 on a coverslip**. (A) Source image. (B) Gradient X image. (C) Gradient Y image. (D) Hue–saturation–brightness (HSB) color-coded map. (E) Distribution of the orientations. The color wheel is also reported.

1. **Ellipses method application to select the HCMEC/d3 actin filaments in BBB model.**

The regions of interest (ROIs) used for the actin filament alignment analysis correspond to the areas circumscribed by ellipses in Figure 5F and 6F of the Main Text. These ROIs were carefully selected to exclude regions where pericytes were in contact with ECs, as indicated in Figure S7. By focusing on these specific areas, we ensured that the actin alignment analysis was primarily quantifying the filaments of HCMEC/d3, minimizing the potential influence of pericyte actin filaments.

**Figure S7. ROIs selection on microchannels of the BBB model.** Ellipses (red) were drawn excluding the regions where pericytes (magenta) were localized and focusing on F-actin (green) of HCMEC/d3. (Scale bar = 50 µm)

1. **Imaging Permeability**

Live-imaging is particularly well suited for studying the permeability of low molecular weight molecules, due to their rapid diffusion dynamics. To facilitate this approach, we developed a smaller “free” chip variant consisting of 12 microchannels aligned with a 3 mm diameter milli-well, offering improved experimental control in real-time analyses.

We tested the chip’s response in the absence of cells (blank chip), but in the presence of Matrigel and culture medium, following the injection of 1 mg/mL FITC–Dextran 4 kDa (Figure S8). Immediately after injection (T1), we observed rapid diffusion of the fluorescent tracer throughout the chip. After 10 minutes (T10), fluorescence intensity increased notably at the membrane—particularly at the microchannel–milliwell interface (highlighted by red rectangles in Figure S8)—likely due to dextran adsorption by the membrane material. This behavior is in line with the high diffusivity of small molecules such as 4 kDa dextran.

This preliminary real-time imaging test, performed without altering the chip setup or disturbing culture conditions, provides the basis for quantifying corrected fluorescence intensity in the presence of cells and determining the actual amount of dextran crossing the interface. Future studies will focus on defining the most appropriate experimental conditions—such as molecular concentration, chip geometry, flow rate, region of interest (ROI) selection, and imaging settings—to enable robust comparison of molecules with different sizes.

**Figure S8. Permeability Imaging of a Blank Chip.** T0 = No dextran injection. The signal is 0, corresponding to the medium's baseline signal. T1 = Immediately after dextran injection (1mg/mL). A rapid diffusion of dextran from the microchannels to the milli-well is observed. The Matrigel imbibition is instantaneous and uniform. The black spot in the upper right of the images is an air bubble. T10 = Ten minutes after dextran injection. An increase in signal is observed at the membrane, specifically at the interface between the two layers (see red rectangle), likely due to dextran absorption by the membrane. Optimizations of protocols to observe the diffusion of low molecular weight dextran are underway.

1. **Resistance Measurements**

Our customized system for chip resistance reading (Figure S9) took inspiration from the work of Ahn and colleagues (3). It consisted of four electrode wires: the I_1_ and I_2_ electrodes in silver (Ag), V_1_ and V_2_ electrodes in silver chloride (AgCl). The current is applied between I_1_ and I_2_ and resistance is measured based on V_1_ and V_2_ readings. The electrode wires were placed in a tip, one for each device ports, filled with culture medium to propagate the electrical signal in a conductive liquid and reduce the noise. Resistance readings provided a reliable basis for comparing various device conditions, including the comparison between devices without cells and those with all cells (Figure S10, Supplementary Table 1).

**Figure S9. TEER reading system.** Ag (I_1_ and I_2_), Ag/AgCl (V_1_ and V_2_) electrode wires connected to a volt-ohmmeter (not shown in the picture), generating 10 μA of alternating current at 12.5 Hz.

Figure S10. Chip raw resistance data under different conditions. Chip filled solely with medium, without Matrigel or cells (“Empty”); Chip filled with medium and Matrigel (“Matrigel”); Chip with brain ECs within microchannels, and pericytes and astrocytes in the milli-well filled with Matrigel (“BBB”).

Supplementary Table 1. Raw resistance data measured using EVOM3. Raw resistance readings (five for replicate, n = 3) of chip filled solely with medium, without Matrigel or cells (“Empty”); chip filled with medium and Matrigel (“Matrigel”); chip with brain ECs within microchannels, and pericytes and astrocytes in the milli-well filled with Matrigel (“BBB”).

|  | EMPTY | MATRIGEL | BBB |
| --- | --- | --- | --- |
| CHIP 1 | 93 | 187 | 217 |
|  | 103 | 198 | 263 |
|  | 113 | 201 | 288 |
|  | 120 | 197 | 261 |
|  | 85 | 171 | 259 |
| CHIP 2 | 88 | 197 | 287 |
|  | 122 | 180 | 251 |
|  | 76 | 179 | 225 |
|  | 15 | 197 | 271 |
|  | 102 | 189 | 285 |
| CHIP 3 | 92 | 195 | 253 |
|  | 115 | 175 | 224 |
|  | 124 | 197 | 269 |
|  | 122 | 173 | 290 |
|  | 100 | 200 | 263 |

1. **Flow Frequency Measuring.**

Pulsating flow frequency quantification was carried out using a commercially available flow sensor (MFS2 by ELVEFLOW), operating within the 0 – 7 μL/min range with an accuracy of 0.02 µl/min. The chosen flow rates for the experiments were: 0.025, 0.05, 0.1 µL/min, akin to those employed during the culture period of brain ECs within the microchannels (specifically 1.5 µL/h, corresponding to 0.025 μL/min). Consequently, the plots depicting flow rate variations over time revealed pulsating flow with a frequency ranging from 0.5 to 1 Hz (Figure S11).


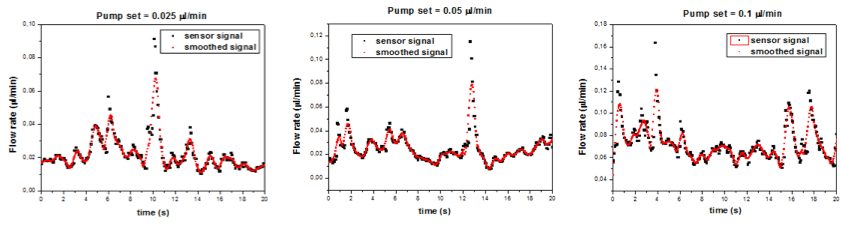

Figure S11. Flow rate variations over time. The plots show the time-dependent flow rate (displayed in 20-second intervals) for syringe pump flow rate settings of 0.025, 0.05, and 0.1 µL/min. In all cases, oscillations occur approximately once every 1 or 2 seconds, corresponding to frequencies of 1 Hz or 0.5 Hz, respectively.
